# Modal and amodal cognition: an overarching principle in various domains of psychology

**DOI:** 10.1007/s00426-023-01878-w

**Published:** 2023-10-17

**Authors:** Barbara Kaup, Rolf Ulrich, Karin M. Bausenhart, Donna Bryce, Martin V. Butz, David Dignath, Carolin Dudschig, Volker H. Franz, Claudia Friedrich, Caterina Gawrilow, Jürgen Heller, Markus Huff, Mandy Hütter, Markus Janczyk, Hartmut Leuthold, Hanspeter Mallot, Hans-Christoph Nürk, Michael Ramscar, Nadia Said, Jennifer Svaldi, Hong Yu Wong

**Affiliations:** 1 Department of Psychology, Fachbereich Psychologie, University of Tübingen, Schleichstr. 4, 72076 Tübingen, Germany; 2 Department of Psychology, University of Augsburg, Augsburg, Germany; 3 Department of Computer Science, University of Tübingen, Sand 14, 72076 Tübingen, Germany; 4 Leibniz-Institut für Wissensmedien, Tübingen, Germany; 5 Department of Biology, University of Tübingen, Auf der Morgenstelle 28, 72076 Tübingen, Germany; 6 Department of Psychology, University of Bremen, Bremen, Germany; 7 Department of Philosophy, University of Tübingen, Tübingen, Germany; 8 German Center for Mental Health (DZPG), partner site, Tübingen, Germany

## Abstract

Accounting for how the human mind represents the internal and external world is a crucial feature of many theories of human cognition. Central to this question is the distinction between modal as opposed to amodal representational formats. It has often been assumed that one but not both of these two types of representations underlie processing in specific domains of cognition (e.g., perception, mental imagery, and language). However, in this paper, we suggest that both formats play a major role in most cognitive domains. We believe that a comprehensive theory of cognition requires a solid understanding of these representational formats and their functional roles within and across different domains of cognition, the developmental trajectory of these representational formats, and their role in dysfunctional behavior. Here we sketch such an overarching perspective that brings together research from diverse subdisciplines of psychology on modal and amodal representational formats so as to unravel their functional principles and their interactions.

How humans mentally represent information is a fundamental issue within psychology and beyond. Not surprisingly, most theories about human cognition involve representational assumptions, at least implicitly. Depending on the domain of investigation, different types of mental representations are typically in the foreground. In research on thinking, memory, or language processing, the traditional assumption is that properties, objects, situations, and events are captured through symbolic representations (e.g., Evans et al., 1993; Fodor, 1975; Kintsch, 1998; Pylyshyn, 1984; Quilty-Dunn et al. 2022; Reed, 2016; Smith & Medin, 1981; Tulving, 1972). These symbolic representations typically do not resemble a specific state of affairs as it stands. Instead, they abstract from details of the situation, allowing for categorization of the things they represent (e.g., dog vs. cats). For instance, the meaning representation of a word such as “dog” must somehow encompass features of very different types of dogs and can thus be considered an abstract representation. In addition, symbolic representations are usually considered to be independent of the characteristics of any particular sensory modality. In other words, although many of these representations emerge from sensory experiences, these experiences are no longer part of the resulting representations. For example, although the meaning of the word “melody” mainly will refer to auditory features, the representation of this meaning within the mental lexicon will no longer encompass a specific auditory experience because it is more abstract than any particular experience. Likewise, although the word “stain” mainly refers to visual features, its symbolic meaning representation will itself be abstracted from visual experience. It is thus reasonable to assume that these two symbolic representations—for “melody” and “stain”—share a common format, despite them both referring to entities that are typically perceived via different senses. Accordingly, these symbolic meaning representations can be modality-unspecific.

By contrast, in research on perception, it is often assumed that representations are modality-specific and resemble the entity being represented. For instance, when perceiving a dog, humans appear to create a rather specific representation that preserves many of the properties of the particular dog being perceived. In this sense, the representation is concrete rather than abstract. From this perspective, perceptual representations can be seen as being inherently different, depending on whether the represented entity is mainly characterized by auditory, visual, olfactory, or other sensory features. Accordingly, these representations can be modality-specific. This is also a core assumption in various theories on imagery, which suggest that mental representations are concrete and modality-specific (e.g., Kosslyn, 1980; but see Pylyshyn, 1981, and also Pitt, 2013) and thus are quasi-perceptual (Ward et al., 2019).

However, researchers in most domains of cognitive psychology nowadays do not assume that all mental representations are of one exclusive type. For instance, in research on conceptual knowledge (for an overview, see Murphy, 2002; Pecher, 2013), hybrid forms of mental representations are explicitly discussed. For example, a hybrid representation of the concept 'dog' could consist of a symbolic component listing typical attributes of dogs and also a component with experiential traces that stem from sensory experiences encountered in the past. Finally, it is worth noting that the two representational formats described above are unlikely to constitute a dichotomy in a strict sense. Instead, they may represent the endpoints of a “continuum” that ranges from modality-specific to abstract symbolic representations (see Fig. 1; Meteyard et al., 2012; see also Gentner & Asmuth, 2019). We deliberately placed "continuum" in quotation marks because the transition from concrete to abstract might not be entirely continuous but may contain a discontinuity. This seems especially true when it comes to a transition to propositional representations with symbols as elements (see the transition between the last two representations in Fig. 1 marked by a dashed line to indicate the possible discontinuity).

**Fig. 1:**
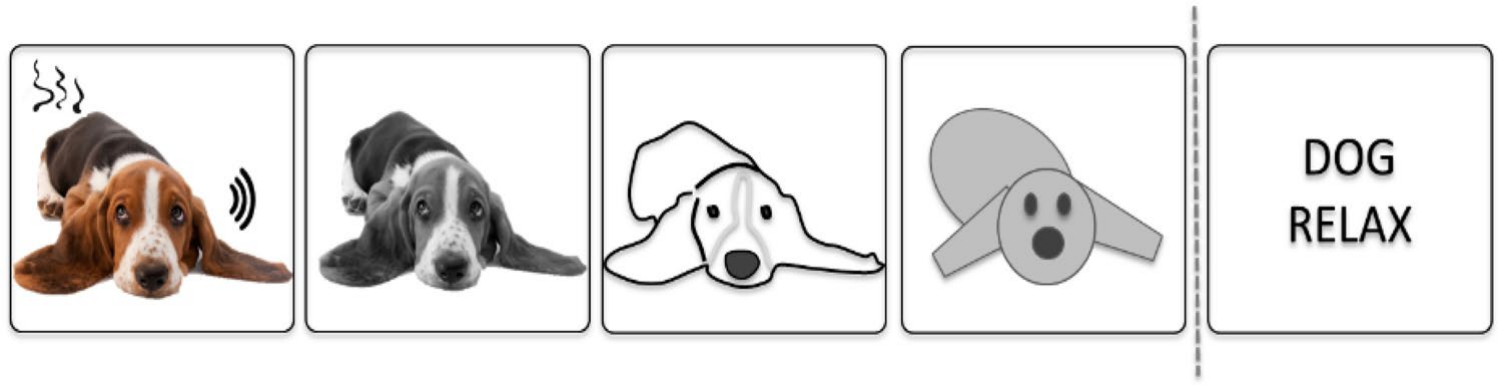
Illustration of the continuum ranging from very concrete modality-specific representations on the one hand (left side) to more abstract symbolic representations on the other hand (right side)

In summary, questions concerning the nature of mental representations are central to virtually all domains within cognitive psychology, and different types of mental representations are explicitly discussed in many of these domains. Nonetheless, an overarching analysis of representational issues, particularly concerning various types of representations, is to the best of our knowledge, not yet available. Consequently, it seems that cognitive psychology lacks a comprehensive theoretical account of the functions and interactions of different representational formats, thus remaining poorly understood. Needless to say, it follows that the same can be said of the domains of application, developmental trajectories, and possible malfunctions of these representational formats. Moreover, computationally it is something of a given that the nature of a cognitive process will depend on the format of the representation it operates on (Bröker & Ramscar, 2020); as is the reverse, namely that particular processes might call for particular representational formats (e.g., putative combinatoric processes in language might require more abstract proposition-like representations, whereas processes utilized for immediate action might require very detailed representations in absolute metrics (e.g., Ganel & Goodale, 2003). Thus, a comprehensive understanding of cognitive processes would also appear to require a better understanding of representational format. 

We suggest that one goal of current research in cognitive psychology research should be to better understand representational issues in cognition. We consider it theoretically fruitful to assess whether it is possible to integrate the various representational formats from different subfields of cognitive psychology (e.g., propositional representations vs. mental models in research on spatial reasoning; prototypes vs. exemplars in research on conceptual knowledge; cf. Murphy, 2016). We suggest that it is worthwhile to investigate whether all representational formats can be located on one continuum from concrete to abstract. Such a research endeavor would not only allow parsimonious explanations of the respective phenomena but also allow for theoretical relationships between the different subfields of psychology to be uncovered, paving the way for a more general theory of human cognition.

One factor that complicates this endeavor is that a wealth of different terms are used to refer to the respective representational distinctions: *concrete* vs. *abstract* (Reed, 2016; Snodgrass, 2006), *symbolic* vs. *analog* (Dehaene et al., 1998; Furman & Rubinsten, 2012), *propositional* vs. *analog* (Johnson-Laird, 1983; Zimmer, 2006), *digital* vs. *analog* (Dretske, 1981; Katz, 2008), *perception-based* vs. *meaning-based *(Anderson, 1995*)*, *descriptive* vs. *perceptual* (Newen & Marchi, 2016), *modality-specific* vs. *modality-unspecific* (Vaina, 1984), *modality-specific* vs. *supramodal* (Binder & Desai, 2011; Kiefer & Pulvermüller, 2012), *perspective-specific* vs. *perspective-flexible* (Brunyé et al., 2008), *LoT-like* vs. *non-LoT-like* formats (Quilty-Dunn et al., 2022), to name just a few. One pair of terms that recently gained much attention in research on cognitive psychology is the opposition between *modal* and *amodal* representations (Anderson, 2009). In the following, we will base our considerations on these terms in the following way (see Fig. 2).^1^

**Fig. 2:**
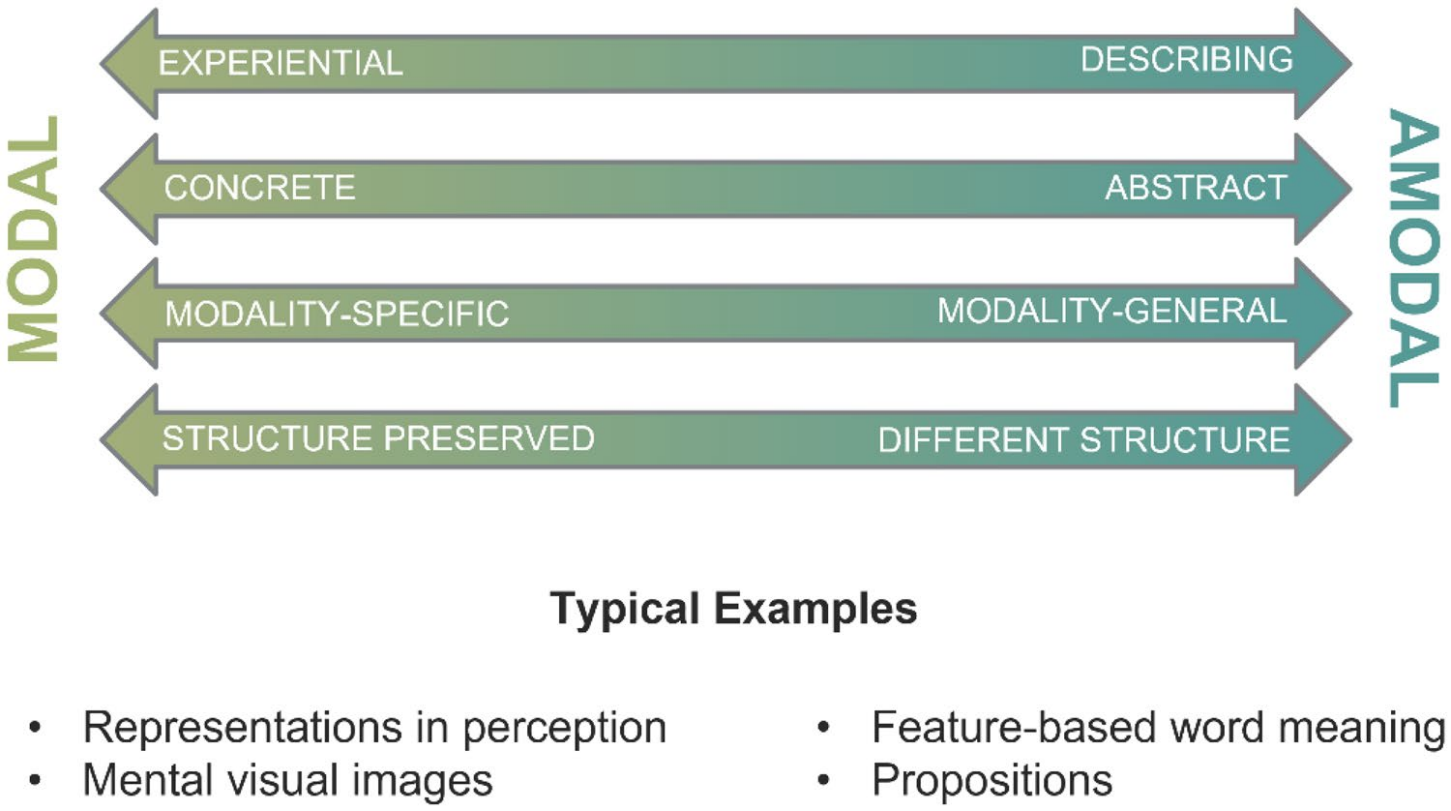
Properties and examples of modal and amodal representations (see text for further explanations)

*Modal representations* are fundamentally experiential in nature and are therefore rather concrete. The structure of these representations preserves structural aspects of how we experience the world: that is, mappings between the world on the one hand, and representations of it on the other, are isomorphic. Sensory representations in perception that rely on prothetic continua^2^ (e.g., intensity) or involve mental images are classical examples of modal representations. As these examples suggest, modal representations can be relatively simple (e.g., representing one particular value of an attribute dimension) or highly complex (e.g., representing a rich image of a multi-faceted situation). Moreover, modal representations need not necessarily concern only one sensory modality, but may draw on several different modalities (for instance, by means of cue integration, e.g., Ernst & Banks, 2002). Information from various modalities may be associated in a modal representation (e.g., the smell and the sound of a dog), but, importantly, in a modal representation, there is no representational component that combines information in an abstract, modality-unspecific way. Instead, modal representations either concern only one modality or encompass several modality-specific representations (for one idea of how the individual modality-specific representations can be bound together, see the convergence zone framework proposed by Damasio, 1989). Modal representations are often also considered holistic rather than compositional (but see Barsalou et al., 2003).

*Amodal representations,* by contrast, encompass an abstract description of the state of affairs they represent. Their structure is different from the structure of the things they represent. Amodal representations may capture information from one or more modalities, but these representations themselves are modality-unspecific. Feature-based word meaning representations, semantic networks, schemata, and frames are examples of amodal representations. Propositional representations constitute another typical example. Traditionally, these representations are held to be symbolic codes emerging from combining elementary building blocks of meaning. These representations can be combined or “composed” into more complex propositional representations in much the same way words are combined into sentences (Frege, 1892). Thus, like language, propositional representations are held to be compositional. In this sense, propositional representations are often considered to be linguistic representations and are usually supposed to be the language of thought, according to traditional theories in cognitive science (Fodor, 2008; Pinker, 1999). Like modal representations, amodal representations can be simple (e.g., capturing only a single attribute or entity) or highly complex (e.g., capturing a series of events and situations interconnected by causal relations and involving many different objects and attributes). Although propositional representations are certainly a prime example of amodal representations, amodal representations need not necessarily be language-like. For instance, a representation of an object in terms of elementary geons (cf. the recognition-by-components theory of Biederman, 1987), as opposed to several viewpoint-specific holistic object representations (cf. Tarr & Bülthoff, 1995), is non-linguistic but shares several aspects of amodal representations (e.g., a finite set of basic components, compositional structure, view-point invariance). Thus, Geon theory is closer to the amodal end of the modal-amodal continuum than, for instance, a visual image of an object (see Fig. 3). In addition, although proposals for amodal representations with a compositional structure seem particularly suited to accounting for meaning representations in language and other higher-level cognitive processes (such as reasoning or problem-solving), they have also been postulated in other domains of cognition, such as in action planning (e.g., Glover, 2004).

**Fig. 3:**
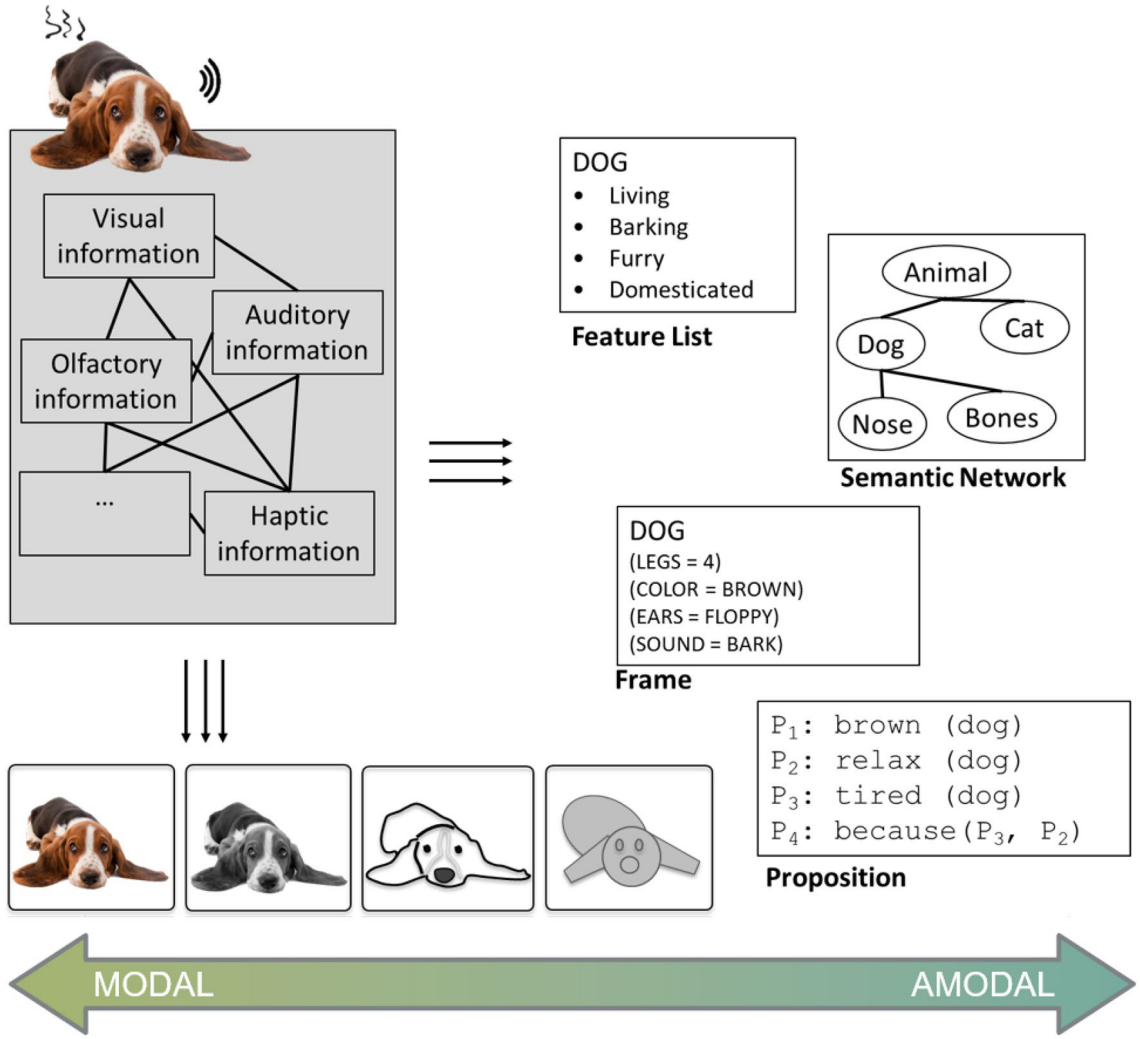
The modal-amodal continuum comprising different forms of representations, ranging from image-like representations to frames and propositions

It is also important to point out here that not all authors agree with the above characterization of linguistic meaning representation in terms of a strictly compositional rule-based assembly of discrete elementary building blocks which is intrinsic to the traditional Language-of-Thought/Generative-Grammar Paradigm in cognitive science (Fodor, 1975, 2008; Fodor & Pylyshyn, 1988). Increasingly many authors instead assume meaning representations of different levels of abstraction that are grounded in perception and action, and are characterized by weak compositionality (e.g., Goldberg, 2003, 2019; Langacker, 2008; see also Michel, 2023).

The distinction between modal and amodal representation applies not only to information input but also to action. For example, a motor plan can be specific to a particular muscle group or a limb, which would be a modal representation of a concrete action (e.g., pointing with the right index finger to a target object). This notion resembles the motor plans suggested by Keele (1968). However, others like Schmidt (1975; see also Rosenbaum, 1980) assume that each amodal motor program is represented as a schema. For example, such a schema would allow one to produce one’s signature even with different effectors (e.g., the fingers when signing a check or the whole arm when writing the same signature much larger on a blackboard; see Liu et al., 2020, for recent neuropsychological evidence for effector-independent action representations). 

Our distinction between modal and amodal representations is largely consistent with the instance level of Reed's taxonomy of abstraction (Reed, 2016), according to which the terms modal vs. amodal refer to representations of instances, such as the representation of a particular dog. A modal representation is a concrete representation that resembles an earlier sensory experience that can be activated without external stimulation. By contrast, amodal representations are abstract, and under some accounts extend to including propositional representations of meaning, in which a proposition is anything that can be asserted or denied using words (e.g., my dog smells bad) and can be determined to be true or false (Reed, 2016). Therefore, according to Reed, amodal representations can be evaluated for their truth value and modal representations for their similarity.

Above, we specified several attribute dimensions on which modal and amodal representations may differ. The question arises whether these dimensions are correlated and whether a particular dimension is more prominent than others. We assume that two dimensions are particularly crucial and thus define our framework. One dimension runs from structure-preserving to structure-agnostic, or in other words, from analog to symbolic. The other dimension runs from modality-specific to modality-general, whereby modality here refers to perceptual modality (visual, auditory, tactile, etc.) or response modality (arms, feet, mouth, etc.; see Fig. 4). We believe that the distinction between modal and amodal representations is best captured by the diagonal in this diagram. Thus, the prototypical modal representations are located in the lower left and the prototypical amodal representations in the upper right quadrant. For example, the multiple-views theory assumes several canonical view-point specific holistic object representations that are rotated to align with the input image during object recognition (Tarr & Bülthoff, 1995) and can thus be located in the lower left quadrant. Likewise, a prime example for a theory in the upper right quadrant are views postulating propositional representations created during language comprehension which are truly symbolic and capture information from different modalities in a modality-general way (Kintsch, 1998; McKoon & Ratcliff, 1992). Another less prototypical but at least as clear example for this quadrant are theories in time perception where time is represented by the number of pulses registered by an internal timing mechanism during the perception of a time interval (e.g., Ulrich et al., 2022). This mechanism is not modality-specific as it potentially receives input from different modalities (time intervals during visual or auditory or tactile perception), allowing the comparison of a tone's duration with a light’s duration. Although these are the most relevant quadrants for the distinction between modal and amodal representations, it is nevertheless possible to find examples that fit in one of the other quadrats. For instance, we would locate the Geon theory in the lower right quadrant because this theory assumes visual objects to be composed of geometric ions (e.g., cylinder and brick Geons; Biederman, 1987). For the upper left quadrant, we see Baddeley’s visuospatital sketchpad as a possible candidate, because here representations are analog in nature but not truly visual. Rather they are thought to combine information from visual, tactile and haptic sensory channels (Magnussen, 2013). In summary, we believe that nearly all representational formats can be located within this two-dimensional plane even though it may sometimes be that the suggested format cannot be exclusively located within a single quadrant.^3^

**Fig. 4:**
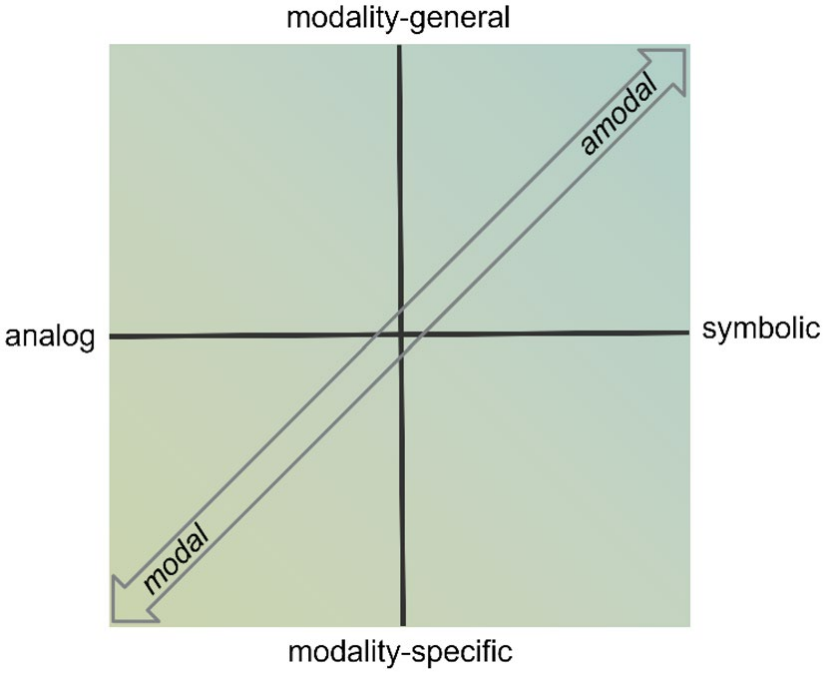
The modal-amodal continuum in a plane given by the two dimensions “analog-to-symbolic” and “modality-specific-to-modality-general”. *Modality may refer to the input side as well as the response side*

The considerations above reveal how the psychological reality of different types of representational formats has been largely accepted in cognitive psychology (albeit this acceptance is typically implicit). It is further notable that this view has recently even found its way into related disciplines such as philosophy of mind (e.g., Butterfill & Sinigaglia, 2014; Camp, 2009; Wajnerman Paz, 2018) and cognitive neuroscience (e.g., Leshinskaya & Caramazza, 2016, see also Kuhnke et al., 2022). Yet it remains the case that the relationship between these formats and their functions for cognition is intensively debated, especially in the literature on the human conceptual system (e.g., Barsalou, 2016). In fact, the theoretical accounts range from a strong view of grounded cognition, which assumes that concepts are modal representations (e.g., Glenberg & Gallese, 2012) to a view that assumes that concepts are represented in an amodal format (e.g., Machery, 2016; Mahon, 2015). However, most accounts consider a hybrid view according to which the cognitive system involves both representational formats (e.g., Binder & Desai, 2011; Dove, 2009, 2022; Kiefer & Pulvermüller, 2012; Zwaan, 2014).

The fragmented debate about the role and functions of modal and amodal representations, along with the many implicit assumptions many theories make about them, calls for an explicit, overarching approach that addresses this issue from different angles within psychology (i.e., perception, action, learning, emotion, language, and thought) and brings together the various theoretical ideas about the interaction and function of amodal and modal representations. In order to arrive at a comprehensive understanding of cognition, these different perspectives on the distinction between modal and amodal representations need to be integrated. A synthesis is required beyond studies within isolated, individual subfields of cognition. Crucially, such an overarching approach would also allow to distinguish between domain-specific and domain-general aspects of the cognitive processes that operate on modal and amodal representation. Accordingly, we consider it of central relevance for research in cognitive psychology to investigate the functions of modal and amodal representations for human cognition and to analyze their interplay within the subfields of psychology.

In what follows, we will briefly sketch the questions arising in the different subfields of psychology concerning the functions and interactions of modal and amodal representations. We start with the subfield of perception, and in subsequent sections discuss the topics of action, cognitive control, learning, emotion, language, thought, development, and dysfunction.

## Perception

Philosophers and psychologists have long speculated about how people perceive the outside world. Extreme positions can be distinguished and classified within the framework mentioned above. It is often assumed that perception comprises elementary units which are assembled or synthesized, a general assumption consistent with the amodal view. One appealing aspect of this view is that it is compatible with a more widespread research strategy, that of decomposing complex systems into their elements, thereby decreasing their complexity, and making research appear more tractable. The underlying assumption here is that the elements identified are functionally independent, and thus can be studied in isolation (Bechtel & Richardson, 2010). This research strategy of decomposing complex systems is evident in cognitive research in general but particularly prominent in the study of perception.^4^

For example, the philosopher John Locke (1632–1704) assumed that complex ideas (e.g., the idea of an apple) are composed of impressions (i.e., sensations, e.g., the features “red” and “round,” and “juicy”) that emerge from the senses (see Hergenhan, 2009). Furthermore, he assumed that these atoms could be combined in an almost infinite number of ways. A similar idea was held by Wilhelm Wundt (1832–1920), who believed that perception is a passive process fed by many simultaneously active elementary primitives. Wundt’s elementism, however, was later challenged by Gestalt psychologists, who coined the well-known phrase “The whole is more than the sum of the parts” and thus tried to understand perception within a holistic framework, a position that is consistent with the modal view.

This basic distinction between holistic and compositional representation is also found in more recent theories of perception. For example, Neisser (1967) in his seminal book *Cognitive Psychology*, elaborated on the distinction between “template-matching” and “feature-analyses” to understand human pattern recognition, which at the time was inspired by the burgeoning field of artificial intelligence. Unlike Gestalt psychologists, who never moved away from the concept of “template-matching” to understand how humans recognize a letter such as A, pioneers of machine perception such as Selfridge (1959) assumed that features are first identified from a pattern resulting in a feature list, which is then synthesized in a subsequent hierarchical process. The basic idea of feature analysis and subsequent synthesis is also found in McClelland and Rumelhart's (1981) interactive activation model or in the Geon theory mentioned earlier (Biederman, 1987). Moreover, psychophysicists have assumed that even low-level processes assemble many spatial frequency codes (i.e., spatial “atoms”, Campbell & Robson, 1968) to account for fundamental phenomena like the perception of Mach band patterns. Likewise, Miller and Ulrich (2003) assumed that basic perceptual processes involve many grains (i.e., temporal “atoms”) to account for various basic findings on reaction time. By contrast, viewpoint-specific theories (e.g., Ullman, 1989) reinforce the notion of “template-matching.” They proceed from the core assumption that each object is represented in memory by a single two-dimensional canonical view to which objects in the three-dimensional view are aligned to for recognition.

Although these two global approaches to object perception (template-matching vs. feature-analysis) are incompatible, each approach can explain certain phenomena that others cannot (Palmer, 1999). Of course, on the flipside, each approach has its own problems. Similar to the case in physics (e.g., concerning the fundamentally different particle vs. wave-theory of light), traditionally no theory has united both approaches in the psychology of perception.^5^ Rather, the field has traditionally been characterized by the existence of a contrasting framework of fundamentally different theories. However, some recent suggestions may help resolve the debate between template-matching versus feature-analysis. For example, the theory of predictive coding (Clark, 2013; Friston, 2010; Rao & Ballard, 1999) envisions perception as a processing hierarchy in which bottom-up (sensory processing) and top-down processing (expectations) interact, an assumption that was also central for McClelland and Rumelhart (1981) in explaining the so-called word superiority effect.^6^ From a predictive coding perspective, perception is based on a dynamic prediction system that generates downward expectations about sensory input and updates these expectations to minimize the error associated with subsequent predictions. The knowledge used for making predictions is stored at several processing levels within this hierarchy of top-down and bottom-up processing. Knowledge stored at higher levels represents abstract information that does not contain specific features. However, moving downward to the other end of this hierarchy, these representations become increasingly specific in their details. Thus, applying the terminology we outlined earlier, the knowledge represented within this hierarchical structure, from its bottom to its top, can be regarded as a modal-to-amodal continuum (cf. Gilead et al., 2020; Hutchinson & Barrett, 2019; Michel, 2021).^7^

The essential function of perception is to allow organisms to interact with the environment. From this perspective it is understandable why many researchers have focused on the interaction between perception and motor function. Although predictive coding also seeks to understand this interaction (see Hutchinson & Barrett, 2019), several researchers have focused solely on sensor-motor interactions. For example, Wolfgang Prinz has argued that perception and motor processes rely on the same representation (i.e., “*common coding*”; Prinz, 1990, 1997). Consistent with this idea, many researchers argue that actions and action intentions can alter perception (e.g., Proffitt, 2006, Witt, 2011; but see Durgin et al., 2012, and also Firestone & Scholl, 2016). Other researchers, however, argue that conscious perception and sensorimotor processes are based on different processes and representations. For example, within the perception–action model (Goodale & Milner, 2018; Milner & Goodale, 1995), it is assumed that object perception and sensorimotor processing involve different cortical streams (the ventral *vision-for-perception* and the dorsal *vision-for-action* stream). Further, it has been suggested that the representations associated with these two streams employ different formats (e.g., Ganel & Goodale, 2003).

Typically, various sensory modalities (i.e., audition, vision, smell, and touch) contribute to perceptual experience. Despite this, people can also compare information across modalities. For instance, as mentioned above people can compare the duration of a tone to the duration of light and vice versa (e.g., Bratzke & Ulrich, 2019; Ellinghaus et al., 2021). They can even compare the brightness of a visual stimulus to the loudness of a tone and vice versa (Heller, 2021; Stevens & Marks, 1965). This aspect of crossmodal processing has been extensively studied in psychophysics, but the cognitive mechanisms underlying this ability are poorly understood. A typical assumption is that crossmodal matching operates on an amodal representation, such as a common intensity scale in intensity matching (Heller, 2021). However, even the representational format of such a fundamental perceptual quantity is not yet well understood (i.e., how intensity is coded and compared intra- and crossmodally). Nevertheless, with regard to time perception, intramodal timing (e.g., comparing the duration of two successive tones) is typically easier than crossmodal timing, a finding that suggests that modality-specific timing also plays a crucial role in time perception (Ulrich et al., 2006). Accordingly, investigating the cognitive mechanisms underlying cross- and intramodal perceptual processes is an essential step toward a better understanding of the interplay between modal and amodal representations in perception.

Research on perception, especially visual perception, usually focuses on stationary stimulus configurations. However, the visual input that humans typically encounter is temporally structured on multiple scales. Humans must deal with dynamic and transient information representing subsequent events. One prominent theoretical account of this dynamic perceptual process is the event segmentation theory (Zacks, 2020; Zacks et al., 2007). The theory assumes that an interplay of bottom-up and top-down processes guides the perception of dynamic events. The continuous incoming stream of sensory information is segmented into meaningful segments at points of change (Zacks et al., 2009), leading to a structured perceptual representation of each event in working memory, the so-called “working event model”. Although working event models are still close to the sensory input, they already constitute a form of abstraction as they are internally structured and interconnected. Constructing working event models is guided by abstract knowledge in long-term memory—more specifically by amodal event models and abstract event schemata. Thus, from the perspective we have laid out above, event perception in this theory can be characterized as an interplay of modal and amodal cognitive processes at different levels of abstraction.

Interestingly, it has been shown that while memory performance is better for excerpts with than without an event boundary (Huff et al., 2014, 2017; Newtson & Engquist, 1976), dual-task performance is worse at event boundaries. Also, the processing of sensory information seems to be increased at event boundaries compared to during an event (e.g., Huff et al., 2012; Zacks et al., 2020). These findings suggest that elaborate updating processes occur at event boundaries (Huff et al., 2012), possibly focusing mainly on sensory information processing. After perceiving an event boundary, however, participants’ memory for details of the sensory information declines (Gernsbacher, 1985). This finding is in line with the view that updating involves the abstraction of information.

The representational formats associated with various aspects of perception may also depend on the specific contextual setting for other tasks, such as object recognition. For example, objects close to an individual’s personal space may be represented in a more modal format when they become more relevant for actions. By contrast, objects outside their personal space may be represented in an amodal format that only includes a few categorical features of an object. This idea is reminiscent of theories of motor control that distinguish between an early phase of action planning and a later phase of action control that works on more amodal and modal representations, respectively (Elliott et al., 2001; Fuster, 2001; Hommel et al., 2001; Jeannerod, 1986; Milner & Goodale, 1995; Thomaschke et al., 2012; Woodworth, 1899).

In conclusion, at their core, psychological theories of perceptual processes have always revolved around representational formats. For this reason, the theoretical relevance of representational formats for understanding cognitive processes becomes particularly prominent in the study of perception. However, contemporary theories of perception have abandoned the traditional dichotomy between modal and amodal representations in favor of a hierarchical view. Here, the assumption is that representations become more abstract (i.e., move from modal to amodal) at higher levels of the processing hierarchy. For example, this view suggests that at higher levels of processing, people can compare information (e.g., stimulus intensity and stimulation duration) across sensory modalities.

## Action

As mentioned earlier, it is often assumed that actions proceed in two subsequent phases: first, relatively abstract aspects of the action need to be decided, such as which effector to use and which type of action to perform. Traditionally, this phase has been called the *planning* phase of the action. Second, concrete details of the selected action have to be specified as, for example, how much force the muscles have to exert to achieve a desired trajectory of the effector or to grip an object, often referred to as the *control* phase (see also Flanagan et al., 1997).

The gradient from relatively abstract planning to fairly concrete control suggests that the internal representations guiding these phases might range from more abstract to more concrete, with the latter including a specific response modality (e.g., left hand). Such a view is in line with the most influential current theories on action control, and ample evidence has been provided to support such a distinction (e.g., Ballard et al., 1997; Bridgeman et al., 1997; Glover, 2004; Goodale, 2020; Hommel et al., 2001; Jeannerod, 1994; Milner & Goodale, 1995; Thomaschke et al., 2012; Woodworth, 1899). Even theories that are mainly concerned with the high-level identification and meaning of actions (e.g., Vallacher & Wegner, 1987, 2012) can be subsumed in this framework because they abstract from details of the implementation of the action (like muscle movements) and can therefore be considered to be working at a relatively abstract level—similar to what other theories call the planning stage of the action.

However, a closer look shows that the evidence supporting the idea of a transition from amodal planning to modal control is not as clear-cut as is often assumed. For example, the theory of event coding (Hommel et al., 2001; see also Janczyk et al., 2023a) and the perception–action model (Milner & Goodale, 1995) currently dominate research on action planning and control. These theories focus on different aspects of actions and usually implicitly presume that representations of different formats underlie them: the theory of event coding is mainly concerned with planning and assumes the underlying representation is amodal and abstracted from modality-specific information. The perception–action model assumes a dedicated sub-system (the dorsal *vision-for-action* stream) that is mainly concerned with controlling actions and operates on modal representations, at least when executing well-practiced skilled actions. Unskilled actions, by contrast, are assumed to be guided by the ventral vision-for-perception stream.

However, while a good amount of evidence supports these postulates, there are studies that might appear to contradict any straightforward distinction between amodal planning and modal control. For example, some studies have indicated that representations of duration and color, that can be conceived as being modal, are involved in action planning (e.g., Koch & Kunde, 2002; Kunde, 2003; see also Kunde, 2006), thereby challenging the idea of (purely) amodal planning. Yet, the empirical picture is not that clear. First, results like those reported by Koch and Kunde might be interpreted as involving some sort of abstraction from the effect colors, which might move the representation more to the amodal endpoint of the continuum. On the other hand, Földes et al. (2008) concluded that the observed effects are due to phonological recoding instead. Results showing that anticipation of the action-effect interval duration prolongs initiation of the action (Dignath & Janczyk, 2017; Dignath et al., 2014) are in line with timing research suggesting that interval discrimination/production can be coded in terms of absolute durations operating on modal representation (e.g., Bartolo & Merchant, 2009; Wright et al., 1997), although more research is needed to test this idea in action control. Likewise, the idea that action planning is based on abstract, amodal representations is not fully compatible with recent studies targeting generalization in response-effect learning and compatibility (see Eichfelder et al., 2023; Janczyk & Miller, 2023; Koch et al., 2021; but see Esser et al., 2023, and Hommel et al., 2003). Other studies have indicated that the execution of unskilled actions is similar to the execution of skilled actions (in terms of Garner interference^8^; see Eloka et al., 2015; Janczyk et al., 2010), in contrast to what is assumed by the perception–action model (e.g., Ganel & Goodale, 2003), further challenging the idea of a qualitative difference between modal (i.e., here: analytical) control of skilled actions and amodal (i.e., here: holistic) control of unskilled actions. In general, the idea that perceptual tasks are influenced by Garner interference whereas motor control tasks are not, is much less well-supported than was initially thought (Bhatia et al., 2022a).

Other studies also undermine the assumption that two fundamentally different types of representations are involved in perception and action control (see the Section on Perception). For instance, it has been claimed that certain visual illusions that are present in perception are not present in grasping (e.g., the Ebbinghaus-Illusion; Aglioti et al., 1995). However, there is good evidence that the Ebbinghaus-Illusion does in fact affect grasping to a similar degree (Franz & Gegenfurtner, 2008; for a multi-lab replication study, see Kopiske et al., 2016). Finally, many studies have claimed to show evidence that while perception follows Weber's law,^9^ grasping does not (e.g., Ganel et al., 2008). However, a recent evaluation of the literature by Bhatia et al. (2022b) calls this claim into question, arguing that when analyses are corrected for methodological problems, grasping follows Weber's law just as perception does. In conclusion, there are several empirical findings that call into question whether it really is the case that early phases of action planning are based on more amodal representations and later phases of action control on more modal representations. Accordingly, further research is needed to understand the different functions of modal and amodal representations, along with their possible interactions in action planning and execution, especially in relation to the time course of actions themselves. For instance, it is possible that secondary tasks involving more amodal representations influence particular aspects of early action phases, whereas secondary tasks involving more modal representations influence particular aspects of later action phases.

## Cognitive control

Cognitive control allows humans to act in a goal-oriented manner according to their long-term plans and to flexibly adapt their behavior to the specifics of situations. Thus, cognitive control (or executive control) is a vital component of the human cognitive system, and two different control modes may be distinguished (Braver, 2012): Proactive control actively maintains goal-directed information to optimize the cognitive system for a forthcoming, demanding event. Reactive control depends on detecting processing conflicts, such as in the Stroop task, where participants experience interference from a word’s meaning when they are asked to report the competing color of the ink it is printed in. Whereas proactive control leads to long-term processing adjustments through top-down biases, reactive control is a transient process triggered by bottom-up processes that leads to processing adjustments in the short-term.

Representational issues are inherent to this distinction between two control modes and are crucial for research on cognitive control in general. Following insights into the functional organization of the prefrontal cortex (e.g., Koechlin et al., 2003; Miller & Cohen, 2001), and in line with the predictive coding view of cognition (e.g., Gilead et al., 2020), it has been suggested that cognitive control is hierarchically structured, operating on concrete stimulus–response associations at the lower end to more and more abstract and domain-general mechanisms and representations at the higher end of the hierarchy (Badre & Nee, 2018; Schumacher & Hazeltine, 2016). For example, the conflict monitoring hypothesis (Botvinick et al., 2001) assumes that conflicting responses developing at lower processing levels are monitored and that an abstract signal (i.e., Hopfield energy) reflecting conflict strength is issued to higher processing levels that bias subsequent information processing.^10^ In contrast to the conflict monitoring hypothesis, episodic memory or binding accounts of cognitive control (e.g., Hommel et al., 2004) assume that reactive conflict adaptations result from the processes operating on specific stimulus–response links (e.g., Frings et al., 2020). Accordingly, these theories predict adaptations specific to individual stimulus–response associations.

There are two main experimental approaches to studying cognitive control effects on performance (e.g., Bausenhart et al., 2021; Dudschig, 2022b), resulting in either local or global processing adaptations. The local approach investigates changes of control on a trial-by-trial basis. Specifically, if an incongruency (i.e., conflict) is detected in one trial, this results in the upregulation of control. Thus, in the following trial, the influence of task-irrelevant information is reduced (see also Botvinick et al., 2001), resulting in a reduced congruency effect. The opposite holds for trials following congruent trials (i.e., non-conflict trials). This adaptation is often referred to as the congruency sequence effect (CSE or Gratton effect; Gratton et al., 1992; Stürmer et al., 2002; for a review, see Braem et al., 2014). The second approach focuses on global instead of local changes in conflict adaptation that may mainly result from proactive control. Specifically, this approach varies the relative frequency of congruent and incongruent trials within a block, and the congruency effects are generally relatively small when the proportion of incongruent trials is high, a finding that has been interpreted in terms of strategic top-down adjustments within information processing (for a review, see Bugg, 2017).

Most importantly, both local and global effects allow researchers to address the distinction between modal and amodal representations by asking whether conflict adaptation is domain-specific or domain-general. For example, most studies report reduced or absent CSEs (as a measure of reactive control) when stimulus and response features or tasks change from one trial to the next (Braem et al., 2014; Dignath et al., 2019), which supports the idea of modal stimulus-response representations underlying the CSE and reactive control. However, a few studies suggest that under specific circumstances domain-general adaptation (operating on amodal representations) can occur (e.g., Hsu et al., 2021; Kan et al., 2013) For instance, when experiencing a syntactic conflict during sentence comprehension, the congruency effect in a subsequent Stroop task is reduced (Kan et al., 2013). Yet, recent attempts to directly replicate this original finding have failed (Aczel et al., 2021; Dudschig, 2022a) and extensions to semantic rather than syntactic conflict conditions were also unsuccessful (Simi et al., 2023). Interestingly, for proactive control, there is some evidence for domain-general adjustments when providing explicit information about upcoming congruency (e.g., conflict) (Bugg & Smallwood, 2016; Jiménez et al., 2021), but overall, not enough research has addressed these important issues for firm conclusions to be drawn.

To sum up, representational issues are of central importance for cognitive control theories. For local conflict adaptation, current evidence suggests that adaptation is based on more modal processes and representations. Proactive control, on the other hand, is probably based on more amodal processes and representations. However, future research is needed to corroborate these representational assumptions in research on cognitive control using paradigms that explicitly address whether variables higher up the hierarchy (e.g., task goals, situational context) implement more abstract representations than the apparently low-level associative stimulus-response mechanisms involved for instance in the CSE. At the same time, to study amodal representations, future research should minimize the contributions of concrete stimulus-response links (cf. Braem et al., 2019; Schmidt, 2019). Finally, it might be worthwhile to employ research methods targeting different representational processing levels. For instance, low-level response activation could be assessed by using mouse-tracking (Potamianou & Bryce, 2023), response force (Weissman, 2019), as well as brain-based measures such as the lateralized readiness potential (Dudschig & Kaup, 2020; Leuthold et al., 2004), which could be complemented by brain-based analysis methods that are specifically suited to reveal also higher-level representational brain states involved in cognitive control (cf. Freund et al., 2021).

## Learning

One of the most basic types of learning is that of learning to represent associations between objects and events (and their attributes) in the world. This process involves learning to discriminate informative from uninformative environmental relationships by means of error-driven processes (Rescorla, 1988; see also Ramscar et al., 2013a, b; Dayan & Berridge, 2014). The notion of associations plays a central role in several domains of cognitive psychology, such as in research on concept learning and semantic memory (e.g., Kelter & Kaup, 2012; Love et al., 2004), word learning (e.g., Ramscar et al., 2013a, b) and conditioning (e.g., De Houwer et al., 2001; Hütter, 2022). However, although the term “association” has a long history, going back to Aristotle and Locke (see Strube, 1984), and is used to explain many phenomena in psychology, it is unclear whether associative learning involves the same types of representations in all cases. In particular, it is unclear to what degree associative learning involves abstraction from individual experiences and, accordingly, the degree to which it involves forming relationships between amodal rather than modal representations is also unclear. In addition, questions about the degree to which abstraction processes are involved in associative learning across different domains, and whether they differ between them, remain largely unexplored.

One way to study these questions is to investigate the factors that trigger abstraction processes and the creation of amodal representations during associative learning. It has been suggested that abstraction is triggered by variability in the exemplars on both sides of the relationship (e.g., Ramscar et al., 2010; Raviv et al., 2022). For instance, if a person sees many different female faces and these are always combined with one of several pleasant images, then this might ultimately trigger the learning of an association between two amodal representations (‘female’—‘positive valence’; Hütter et al., 2014). By contrast, if the person experiences only one specific exemplar on both sides of the relationship, then this is more likely to give rise to the learning of an association between two concrete modal representations (e.g., a visual representation of the face and a concrete positive image). Differences concerning the variability of the exemplars on both sides of the target relationship might explain differences in the stability of the learned associations as they are typically observed in classical vs. evaluative conditioning^11^ (Hofmann et al., 2010). Recent research directly manipulating the variability of the exemplars presented as conditioned stimuli in an evaluative conditioning paradigm showed the hypothesized relationship between variability and abstraction (Reichmann et al., 2022).

One parsimonious explanation for why variability in the conditioned stimuli leads to abstraction begins with the observation that associative learning can be characterized as an error-driven process that serves to reduce learner’s uncertainty about the environment (Rescorla, 1988; Kiefer & Hohwy, 2019). A critical part of the learning process is cue competition, by which the values of reliable cues are reinforced, and unreliable cues devalued during learning (Rescorla, 1988; see also Siegel & Allan, 1996; Miller et al., 1995). As a result, cues that produce little or no prediction error for an outcome will become positively valued at the expense of cues that lead to prediction errors, which become negatively valued (Ramscar, 2021). The result of cue competition is thus discrimination between relevant and irrelevant features, which leads to representations becoming more abstract and amodal (such that Ramscar, et al., 2010 argue that the term ‘associative learning’ is itself a misnomer, and that since in practice, representations in the computational models of this process are largely shaped by the error-driven *unlearning* of uninformative cues, the learning process itself is better conceived of in discriminative terms; for a more traditional perspective, see Reed, 2016 who argues that abstraction processes serve to emphasize distinctive attributes for discrimination). Cue competition is possible in a situation in which a set of complex stimuli predict a set of discrete elements (i.e. when a large cue set is used to predict a smaller set of outcomes, as is the case when a person sees an object with all its features and then hears its label). By contrast, learning from stimuli that lack a rich cue structure hinders cue competition and thus inhibits discrimination learning. For instance, as is the case when a person hears a label and then sees the object it refers to with all its features. Consistent with this, it has been shown that learning to appropriately apply labels to objects is easier for participants when they are trained with a "Feature-Label" procedure compared to when they are trained with a "Label-Feature" procedure (Ramscar et al., 2010). One reason may be that the former leads learners to develop representations that depict the predictive relationships between features and labels, discarding information on non-diagnostic features (i.e., more abstract amodal representations; see also Apfelbaum & McMurray, 2011; Hoppe et al., 2020; Nixon, 2020; Vujovic et al., 2021). The latter may, by contrast, produce representations that provide a more detailed (modal) picture of the structure of the world (i.e., the actual cue probabilities). Thus, there seems to be a trade-off between complexity and discrimination in more abstract amodal representations which seems to be advantageous for labeling (Ramscar, 2013).

However, it is important to note that Ramscar et al.’s (2010) results showed differences in learning outcomes that in principle may or may not be due to different degrees of abstraction in the involved representations. Thus, future research is needed to determine whether learning is advantageous when conditions allow for cue-competition because these conditions lead learners to distort input representations towards abstract representations more than when conditions do not allow for cue competition, and whether these latter situations lead learners to retain more of the modal features of these input stimuli. If this prediction were to be born out in future research, the view that error-driven learning leads to a trade-off between complexity and discrimination could offer a unifying account of the results of studies of the effects of variability on learning and generalization seen across various domains (Raviv et al., 2022).

There may, of course, be other factors that trigger abstraction processes in associative learning. For instance, it has been shown that in concept learning, redundant linguistic labels facilitate the learning process (Lupyan et al., 2007). One reason may be that, as a results of their importance, linguistic labels serve to trigger abstraction processes merely by their presence. In addition, abstraction processes in associative learning might depend on the particular task at hand as well as on the mindset of the learner. It also seems conceivable that the involvement of abstraction processes varies over development. Although grounded cognition researchers often implicitly assume a modal-to-amodal trajectory, there is also evidence for amodal representations being available very early in development (e.g., Rugani et al., 2015; Walker et al., 2010; see also section Development). In our view, examining which types of representations are involved in associative learning under which conditions, and at which points during development, will play an important role in better understanding the functions and interplay of modal and amodal representations. Of course, the nature of the representations involved (modal vs. amodal) is also relevant for other forms of learning, such as procedural or motoric learning (e.g., Pashler & Baylis, 1991a, b), and further investigation in these domains will constitute another fruitful direction of future research.

In conclusion, research into associative learning suggests that variability in the learning exemplars leads learners to build more abstract representations, focusing on the relevant and ignoring the irrelevant stimulus dimensions (cf. Ramscar et al., 2010; Reed, 2016). Also, in concept learning, learners seem to gain from conditions that allow for cue competition and thus in principle provide the opportunity for the learning mechanisms to acquire more abstract amodal representations. However, until now it is not clear whether the gain in performance seen in studies examining this idea is actually due to abstraction processes taking place. Initial research in the domain of evaluative conditioning suggests that this is indeed the case (Reichmann et al., 2022). Future research is necessary to confirm the presumed relationship between learning success and the format of the representations created. Further relevant questions for future research concern not only the domain specificity of abstraction processes, but also their developmental path.

## Emotion

Emotions constitute a fundamental part of human experience, serving several essential functions: emotions drive our actions, communicate relevant information about our internal states to our social surroundings, and guide our attention by informing us about relevant environmental changes. However, theoretical accounts of emotion differ significantly when it comes to the question of representational formats. Some scholars see emotions as an (amodal) memory unit in an associative network where these emotions enter into relationships with coincident events (Bower, 1981). On the other hand, grounded-cognition accounts of emotion postulate a purely modal representational format for emotions (Niedenthal et al., 2005). In contrast to these more extreme views, most accounts assume a hybrid view, acknowledging that emotions have both modal and amodal components. For instance, theories distinguish between ‘hot’ and ‘cold’ aspects of emotions (Metcalfe & Mischel, 1999), feelings and appraisals (Lazarus & Smith, 1988), motor and conceptual level (Leventhal & Scherer, 1987) or between affective and semantic valence (Itkes & Kron, 2019).

Interestingly, when an emotional stimulus is repeatedly encountered (i.e., emotional habituation, Bradley et al., 1993), modal components of emotions seem particularly attenuated. Habituation is assumed to reflect a basic form of memory (Sokolow, 1963). More specifically, theories assume that the current sensory input is compared against a representation of the repeated stimulus in memory: the stronger the match between the two, the more affective responses will be attenuated (Wagner, 1979). Often habituation is linked to changes in attention; after several repetitions, a stimulus provides no further information and attention is allocated elsewhere (Turatto et al., 2018). Under this framework, affective responses are functionally related to attention allocation, because affective responses signal the need for further processing resources (Codispoti et al., 2016; Öhman, 1992). For instance, physiological responses related to valence and arousal are reduced (e.g., Codispoti et al., 2016), as well as behavioral responses (i.e., Jia et al., 2022) and self-reports (Itkes et al., 2017). In contrast, part of the semantic analysis of emotional stimuli, as indicated by the late positive potential in the human EEG, seems to be resistant to habituation (Codispoti et al., 2016). This could suggest that ‘cold’, amodal representations are unaffected by repeated exposure. Such dissociation between ‘hot’ affective responses and ‘cold’ semantic knowledge also is in line with findings of habituation for subjective ratings, suggesting that participants´ self-reports often combine different sources of information: either they focus more on their actual feelings when encountering a stimulus or they can base their judgment more on the semantic knowledge associated with the stimulus (e.g., Robinson & Clore, 2002). One way to disentangle both sources of information are instructions for self-reports that emphasize either feelings or knowledge (Kron et al., 2014). With repeated exposure, feeling-based self-reports habituate, but not knowledge-based self-reports (Itkes et al., 2017). Together, this evidence suggests that repeated exposure influences ‘hot’ and ‘cold’ responses to emotional stimuli differently. While ‘hot’ aspects of affective responses diminish, ‘cold’ aspects of evaluative knowledge remain unchanged. Thus, emotional habituation might be a means to bias processing away from modal and towards more amodal representations in processing emotional stimuli. However, more research is needed to better understand such a representational shift during habituation (see Heimer et al., 2023 for a recent approach).

Another area in emotion research where representational issues seem relevant is the affective priming paradigm (Klauer & Musch, 2003). Here, participants classify the valence of a target stimulus which is preceded by a nominally irrelevant prime stimulus. Evaluative categorizations are faster and more accurate for congruent combinations in which prime and target display the same valence (e.g., happy—sunshine) compared to incongruent combinations in which prime and target display a different valence (e.g., happy—war). Two theoretical accounts have been proposed. The first explanation is based on amodal theories and explains affective congruency effects in analogy to semantic priming (Fazio et al., 1986). The semantic priming account assumes that the valence of the prime stimulus pre-activates a corresponding target valence facilitating responses in congruent combinations, but impairing classification for incongruent combinations. The second explanation is based on theories of response priming, suggesting that a prime activates a corresponding response which then facilitates (or impairs) responses during congruent (incongruent) combinations. The canonical example of response priming are Stroop-like tasks in which relevant target responses compete with irrelevant distractor responses. To explain response priming, theories have adopted a dynamic system view (Scherbaum et al., 2012), which does not employ amodal symbols, but instead describes behavior in terms of continuous states, in line with some (namely anti-representationalist) theories of grounded cognition and simulation (see Spivey, 2008). In fact, Niedenthal and colleagues speculated that “the detection of emotion congruence [in affective priming] is determined by the extent to which the target of judgment must be simulated in order to produce the inference. Extensive simulation will bring more perceptual aspects into the simulation and thereby produce greater, or more easily detectable, emotion congruence. Less simulation will yield a response with fewer perceptual aspects of the concept.” (Niedenthal et al., 2003, p. 316). However, to which extent affective priming reflects ‘hot’ affective responses or rather ‘cold’ evaluative knowledge is a matter of debate (see Rohr & Wentura, 2022). For example, suppose the above considerations are correct and emotional habituation biases towards more amodal processing. In that case, studies examining the effect of habituation on affective priming might be highly informative concerning the psychological reality of the two different types of accounts of affective priming.

In conclusion, although emotions are often characterized by comprising two components one of which is more modal and the other more amodal, it is still unclear how these components interact or under which one or the other component takes the lead.

## Language

According to traditional theories, the representations of meaning involved in language comprehension and production are amodal, and have a compositional structure (e.g., Kintsch, 1988; McKoon & Ratcliff, 1992, 1998; Reed, 2016). During comprehension, people presumably create a coherent network of propositions by identifying the propositions in a sentence or text and their interrelations in terms of argument overlap or rhetorical structure (Asher & Lascarides, 2003; Kintsch & Van Dijk, 1978). Moreover, comprehenders presumably infer particular propositions to fill potential coherence gaps in the linguistic input. Likewise, for language production, such a propositional representation is assumed to constitute the starting point of the production process (e.g., Levelt, 1989). However, grounded comprehension and production models have received more attention within the last two decades. These models assume that modal sensorimotor processes play an essential role in meaning representation during language processing (e.g., Barsalou, 1999; Glenberg & Gallese, 2012; Glenberg & Kaschak, 2002; Zwaan, 2004; for critical reviews, see Machery, 2007, 2010; Mahon & Caramazza, 2008; Winter et al., 2022).

According to these latter models, meaning representations in language processing are assumed to involve the re-activations of experiences with objects, events, and situations that the linguistic stimulus (word, sentence or text) refers to. However, so far, the evidence for this view is mixed. While there is evidence that modal representations are activated during language processing, their activation seems context-dependent (e.g., Lebois et al., 2015; and Yee & Thompson-Schill, 2016 for an overview). Also, it remains unclear whether they play a functional role in language processing (e.g., Montero-Melis et al., 2022; Ostarek & Huettig, 2019; Pulvermüller et al., 2005; Strozyk et al., 2019; Vermeulen et al., 2008; Yee et al., 2013). Thus, it is conceivable that modal representations constitute a mere epiphenomenon, possibly a residual from language acquisition during which sensorimotor meaning representations might be functionally relevant. Alternatively, language comprehension and production might be better characterized by hybrid representations comprising modal and amodal components. This would explain why some studies reported strong evidence for the involvement of modal representations during language processing, whereas others do not (for an overview, see Kaup et al., 2015; Kaup & Ulrich, 2017; see also Berndt et al., 2018, 2020; Ostarek & Hüttig, 2017; Schütt et al., 2022, 2023). Although the hybrid hypothesis has become popular in recent years (e.g., Binder & Desai, 2011; Dove, 2009, 2011, 2022; Wajnerman Paz, 2018; Zwaan, 2014), a systematic investigation of the factors that influence which type of representation gains the upper hand during comprehension and production is still missing.

An important question for future research is to understand better the conditions under which modal and amodal meaning representations play a functional role in language processing. One important factor seems to be the level of processing required by the task; with the increasing likelihood of modal representations, the “deeper” the meaning of the linguistic stimulus has to be processed (e.g., Miller & Kaup, 2020). In addition, the amount and type of direct experiences that a comprehender has made with the described entities and situations in the past determine the type of and the degree to which modal representations are involved in comprehension (e.g., Beilock et al., 2008; Buchanan et al., 2022; Capuano et al., 2023; Casasanto, 2009; Holt & Beilock, 2006; Öttl et al., 2017; Ong et al., 2014; Wolter et al., 2015, 2017). Modal representations seem to be primarily activated if the comprehender has made some form of direct experience with the referents themselves or with a similar referent, allowing for what might be called an “indirect grounding” of verbally acquired concepts (e.g., Günther et al., 2020; Günther et al., 2022; Yee et al., 2013; see also Andrews et al., 2009). Also, as implied by the Construal-Level Theory, one relevant factor might be the psychological distance to the objects, situations, and events that the linguistic stimulus refers to (Trope & Liberman, 2010).

In line with this latter assumption, some recent studies have observed relationships between both temporal and spatial distance and abstractness level (Bausenhart et al., in press; Bausenhart et al., in prep a). More specifically, a series of experiments based on the implicit association test paradigm (IAT, Greenwald et al., 1998; see also Bar-Anan et al., 2006), revealed that response times in a task in which participants decided about the psychological distance a stimulus refers to (proximal vs. distal) or its abstractness (concrete vs. abstract) were influenced by the particular response key assignment in the task. Decisions were faster when distal entities were assigned the same key as abstract entities and proximal entities the same key as concrete entities, suggesting an association between abstractness and psychological distance. Interestingly, for temporal distance, the effects were much clearer when the distal time point to which the “now” (proximal) was compared was the future compared to when it was the past. One potential reason for this is that the future is not only distant but also uncertain, which according to construal-level theory should additionally increase the psychological distance. These experiments show that abstraction level and temporal and spatial distance are cognitively related. However, in the domain of temporal distance, this association seems to be less straightforward and symmetrical than one might expect.

Another series of experiments investigated how spatial distance modulates cognitive representations. Participants were presented with sentence-completion tasks based on a paradigm by Kaup et al. (2021), to assess how distance primes the completion of an incomplete sentence. For example, participants saw an initial sentence fragment that implied near or far spatial distance from the location at which the experiment took place (e.g., *In Los Angeles [vs. Stuttgart], the woman buys…*. They were then asked to select a sentence completion from one of two options, differing in the level of abstraction (e.g., *clothes* vs. *trousers*). In another condition, it was the abstraction level that was manipulated in the initial sentence fragment, and participants were to complete the sentence with the best-fitting spatial location (e.g., *The woman buys clothes in…* to be completed with *Los Angeles* or *Stuttgart*). It was predicted that participants would choose a close location most often for a more concrete term in the initial sentence fragment and a more distal location for a more abstract term, and vice versa. These predictions were clearly born out in certain tasks and conditions, namely when spatial distance was implemented in absolute terms (e.g., by means of explicitly mentioning locations), as well as in a forced-matching task, in which participants were presented both initial fragments and both endings at once and were asked to match them into two sentences. The latter task probably works well because it provides a sort of reference for interpreting the categories. For example, trousers may be specific or abstract, depending on whether they are compared to jeans or clothes (Bausenhart et al., in preparation b).

Another possibility is that cognitive control processes (Botvinick et al., 2001) influence which type of representations constitute the basis for processing. Specifically, it seems conceivable that following an experienced conflict, the linguistic system operates on amodal rather than modal meaning representations. There is little relevant evidence yet with which to evaluate this possibility. The studies mentioned above, looking at the question whether control processes are domain-general or domain-specific did not observe any evidence that perceiving a semantic conflict in one trial of a linguistic task would influence the processing of semantic conflict in a subsequent trial (Simi et al., 2023). One might take this to suggest that conflict adjustments do not target the representational format utilized during language comprehension. However, as these studies did not directly investigate the format of representation, this conclusion is premature. We are not aware of any studies directly investigating the hypothesis of a relationship between the experience of conflict and the representational format used in language comprehension. However, recent studies concerned with the processing of negation may give some hints. Negation has been shown to be difficult to process, and it has been suggested that one reason for this difficulty has to do with the fact that in negative constructions, the non-factual situation is explicitly mentioned (i.e., *The destination is not on the left side*, explicitly mentions the left side although this is the exact opposite of the true destination's property). This might lead to processing difficulties in particular, when comprehenders engage in full-fledged mental simulations of the sensorimotor aspects of the linguistic content, as *not on the left side* would activate sensorimotor processing focusing on the left side (see Kaup & Dudschig, 2020 for an overview on negation research). Indeed, participants show response activation of the contralateral effector when processing phrases like *not left* or *not right* as indicated by the lateral readiness potential (Dudschig & Kaup, 2018). Importantly, however, this tendency to activate the wrong response side following the processing of negation was strongly reduced when the previous trial also contained a negated phrase. This might suggest that the experience of a conflict led participants to reduce simulating the linguistic material and instead turn to more amodal representations that are less prone to automatically activate sensorimotor processes related to the individual words in the linguistic phrases. However, before definite conclusions can be drawn, future research that directly tests the format of the created representations is needed.

In conclusion, there is much evidence that language comprehenders use modal meaning representations during comprehension. However, it is still unclear whether these are functional for comprehension or not. Further, the exact conditions under which comprehenders use more modal or more amodal representations have yet to be determined. Several factors are likely to play a role, including the level of processing required by the task at hand and the amount of experience a comprehender has with the reference entities. Additionally, it seems likely that the psychological distance to the reference situation or the disruptions that occurred through modal processing in previous processing may also play a role. Future research is required to investigate the interplay between modal and amodal meaning representations during language comprehension, and to establish their functional role for comprehension.

## Thought

According to many researchers in cognitive psychology, thinking is deeply rooted in how people perceive space. Space is thus assumed to be an essential component of cognition. Accordingly, space serves to structure thoughts and thus enables humans to understand the world around them. According to metaphoric mapping accounts (Boroditsky, 2000; Gentner et al., 2002; Lakoff & Johnson, 1980; Lakoff & Núñez, 2000, Winter et al., 2015), abstract thinking is achieved by mapping abstract domains that cannot be directly experienced onto modal domains that can be more directly experienced. In particular, it is often believed that spatial experiences structure thinking about non-spatial domains such as time or numerosity (Boroditsky & Ramscar, 2002; Casasanto et al., 2010), allowing reasoning about magnitude. For example, a study by Janczyk et al. (2023b) indicated that although space and time are mentally associated, as are space and numbers, time and numbers are not mentally associated in the same way. This result is consistent with the notion that the non-spatial domains, numerosity and time, draw on spatial thinking.

However, not all authors agree that space is a predominant feature of quantitative reasoning. For instance, Walsh (2003) proposes that humans rely on a general magnitude system, which processes magnitude information regardless of whether it relates to space, numbers, or time (see also Bueti & Walsh, 2009). In contrast to the metaphoric mapping view, this view assumes an amodal representation as the basis of quantitative reasoning (but see Patro et al., 2016b). To summarize, it is controversial whether quantitative reasoning exclusively operates on amodal or modal representations, or a hybrid of both.

For example, the Spatial-Numerical Associations of Response Codes (SNARC) effect describes the associations between smaller numbers with the left side of space and larger numbers with the right side of space observed in Western cultures (Dehaene et al., 1993). Many influences have been demonstrated in SNARC research that are typically explained by referring to modal representations, in particular by assuming that numbers are positioned on a mental number line, which is shaped by experiences (e.g., Fischer & Shaki, 2015; Patro et al., 2016a). However, little is known about how different modal representations interact with space-number associations, because different modalities are usually associated. Some studies provide first evidence concerning different modal influences on the SNARC effect by employing a virtual-reality setup, in which the perceived placement of the hands is manipulated independently of their actual location. These studies suggest that when the numbers are presented close to the body within the reaching space of the hands, the arrangement of the perceived hands in space does not matter much. Instead, the decisive factor in this case seems to be which hand (left or right) is used for responding. By contrast, when the numbers are presented further away from the body and hands, the hands’ arrangement matters, strengthening the horizontal or sagittal SNARC depending on their perceptual arrangement (Koch et al., 2022; Lohmann et al., 2018). In summary, the influence of different modalities for the SNARC seems to depend on the sensory and motor conditions of the setup.

Other accounts seek to explain the SNARC effect by exclusively invoking amodal representations. For instance, the serial order working memory account (e.g., van Dijck et al., 2014) postulates a domain-general mechanism that spatially orders all numerical and non-numerical sequences. In typical SNARC studies, there is a strong correspondence between the ordinal position of numbers and their magnitude, making it difficult to differentiate whether the ordinal position or the numerical magnitude is the crucial factor of the SNARC effect. In a recent study, however, the two accounts were compared directly using different stimulus sets in which the ordinal position of particular critical numbers differed considerably from their magnitude position on a continuous mental number line (e.g., 1,2,3,8; 2,3,4,9; 1,6,7,8; 2,7,8,9). Overall, the response-time pattern obtained with a parity-judgment task requiring left vs. right-hand responses supported the view that number magnitude is mentally mapped to space according to magnitude as well as ordinal sequence. This effect even held when the serial position of the numbers was made salient by having participants learn the serial order of the numbers beforehand and recall the number sets after the parity judgment task. One potential limitation of these result could be that the learned set was irrelevant for solving the parity-judgment task. Thus, follow-up studies are needed to rule out that different results would be obtained when the learned sets are relevant for the SNARC task (Koch et al., 2021).

As mentioned earlier, space is believed to modulate the representational format of human thinking in another research domain. In particular, the construal-level theory postulates that thinking about specific states of affairs involves representations at different levels of abstractness depending on the psychological distances to the state of affairs in question (Trope & Liberman, 2010). However, although previous research has indicated that representations differ for proximal vs. distal things, the cognitive format of these representations has not yet received much attention. First evidence for distance-dependent representational formats was obtained in a recent study on spatial landmark memories. More specifically, LeVinh et al. (2020) investigated the so-called position-dependent recall effect in a virtual environment simulation of familiar places in Tübingen, a small university town in Southern Germany. Participants were immersed in a virtual environment showing a familiar location in downtown Tübingen. After ensuring that the location was recognized, subjects turned until they found a workspace laid out so that they had to take a particular body orientation to complete the task. The workspace comprised five objects identifiable as buildings surrounding a particular target area (in this case, the Timber Market). Participants were then asked to drag and drop the blocks into a configuration rebuilding the target area. The compass bearing of the produced viewing direction was recorded. These compass bearings clearly showed a position-dependent recall effect, meaning that participants built their configuration from the viewing direction consistent with their current location. More importantly for our present purposes, however, the strength of this position-dependent-recall effect decreased with the distance to the target area for two-thirds of the participants.

In conclusion, space seems to play a fundamental role in thinking related to the planning of actions and navigation and for more abstract thoughts such as reasoning about time and number. However, whether these phenomena can best be explained through modal or amodal representations is still debatable. So far only a few research studies have been conducted to understand the interplay between modal and amodal representations in explaining the respective effects. We think that this is a pressing issue for future research.

## Development

The issue of the representation format has only started to become the focus of developmental research. However, the idea that sensorimotor information initially drives ontogenetic cognitive development and thus forms the basis of higher cognitive processes has been around for a while. It was already an important aspect of Jean Piaget’s work (Piaget, 1952). However, Piaget did not argue for strong interactions between modal and amodal cognitive processes. Instead, he suggested that children progress to concepts that are independent of their sensorimotor experiences during their primary school years. Contrary to this assumption, more recent work suggests that perceptual simulation is crucial for the development of higher cognitive processes, even in school-aged children (e.g., De Koning et al., 2017; Engelen et al., 2011; Vogt et al., 2019) and in adults (e.g., Borghi et al., 2004; Pecher et al., 2003; Stanfield & Zwaan, 2001).

**Fig. 5:**
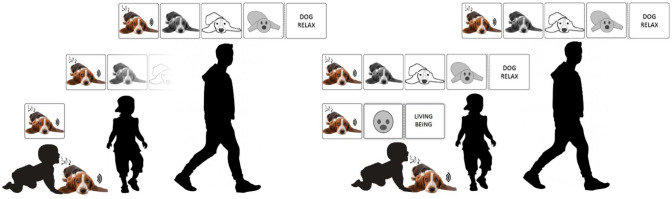
The modal-to-amodal trajectory view (left) versus the early-amodal-representations view (right)

However, one objection must be considered when interpreting these and other similar results. The involvement of modal representations in higher cognitive processes appears to be context- and task-dependent in adults (e.g., Areshenkoff et al., 2017; Bub & Masson, 2010; de la Vega et al., 2012; Dudschig and Kaup 2017; Dudschig et al., 2015, Hoenig et al., 2008; Lebois et al., 2015; Louwerse & Jeuniaux, 2010; Ostarek & Hüttig, 2017; Pecher, 2013, 2018; Pulvermüller, 2013; Ulrich & Maienborn, 2010; Van Dam et al., 2012, 2014; Yee & Thompson-Schill, 2016; Tomasino et al., 2007). Thus, as mentioned earlier, the claim that modal representations in adult higher cognitive processes constitute a mere epiphenomenon without functional relevance cannot be ruled out (Ostarek & Huettig, 2019). One possible explanation for why adult modal representations can nevertheless become activated during higher cognitive processes is that such activations are merely residual manifestations of earlier cognitive development. According to this view, modal representations are functionally relevant for higher cognitive processes early on during development, but they lose their functional relevance during the course of development such that their manifestations in adults are epiphenomenal (see Fig. 5, left side). This view in turn suggests that the cognitive-developmental trajectory proceeds from modal to amodal representations.

Such embodied conceptualizations contrast with recent theorizing based on evolutionary psychology and comparative research (e.g., Spelke & Kinzler, 2007). This line of argument maintains that early cognitive development is guided by abstract core knowledge (e.g., about living beings, objects, actions, numbers, and space), presumably preparing humans to process modal information in a particularly efficient way. Theories and findings on category acquisition might serve as an illustration of different pathways related to the emergence of amodal representations during ontogenetic development (see Fig. 5). According to the theory of Piaget, cognitive psychology initially considered first categories to be basic level ones, based on perceptual similarities (Rosch et al., 1976). However, research with infants showed that early categorization can occur on more than one basis and that basic level categories are often acquired later than particular superordinate categories (Mandler, 2004). For example, infants appear to have amodal representations available in the field of biological understanding, regarding the classification of living beings. Here, children do not build up biological knowledge by learning specific, concrete information and eventually progressing to abstract rules and principles. Instead, they appear to start with an initially amodal abstract core concept of “living beings”, which structures several abstract modality-specific patterns, such as typical visual properties of faces. During development, the system gradually fits in all the concrete details and mechanisms that elaborate those concepts and patterns.

A somewhat comparable perspective also emerged in associative learning beginning with Seligman's findings indicating that abstract visual properties promote specific types of learning (e.g., rapid learning of snake- or spider-fear associations, Seligman, 1970). Interestingly, preparedness has been shown to be already evident in infants, who rapidly establish fear of snakes even when they had no experience of snakes (DeLoache & LoBue, 2009). In other words, in contrast to the modal-to-amodal trajectory proposal mentioned above, other accounts favor the idea that amodal representations are available even early in development. According to this view, rudimentary abstract concepts scaffold the encoding of modal experiences, enriching and restructuring amodal representations (see Fig. 5, right side).

Further relevant evidence comes from studies employing the looking-while-listening paradigm (Bergelson & Aslin, 2017; Bergelson & Swingley, 2012) to investigate the processes underlying vocabulary learning in infants (Kartushina & Mayor, 2019; Steil et al., 2021). In this paradigm, infants hear spoken words and see two pictures on a screen while their eye-movements are recorded. If an infant focuses on an appropriate object when hearing a label, this is taken as evidence that the infant has learned the label. The results of such studies appear to provide further evidence that cognitive processing makes use of amodal information even in infancy. Specifically, it has been shown that the success of infants matching objects to labels correlates with differences in the frequency with which objects occur in their lives: infants correctly fixate on the labeled object more often when the difference in frequency between the two objects shown is higher. This result can be interpreted as showing that infants learn to match objects that they frequently see to labels that they frequently hear, which in turn suggest that children’s associative learning mechanisms are capable of mapping between experiences in different modalities. In terms of our proposed framework, this suggests that the learning mechanisms that lead to the development of abstract, amodal representations are available even at the earliest stages of development.

In addition to these considerations concerning the two different developmental trajectories, research in developmental psychology can also be informative regarding the function of amodal representations for cognition. For example, it is well known that achievements in cognitive development are often correlated with achievements in linguistic abilities (e.g., Schneider et al., 2004). This relation has been particularly intensively discussed concerning the theory of mind abilities (e.g., De Villiers, 2007; Milligan et al., 2007, and the contributions in Astington & Baird, 2005). In principle, these relationships may reflect an underlying development in the ability to use amodal representations (cf. Dove, 2014). Amodal abilities might then boost linguistic abilities (e.g., using terms for mental states) and non-linguistic cognitive abilities (e.g., understanding the mental state of others). If so, studying the relationship between the developing linguistic and non-linguistic cognitive abilities will contribute to a better understanding of the functions of amodal representations for cognitive processes in general.

In conclusion, representational issues seem of great importance to developmental theories. In principle, two different perspectives can be outlined that postulate either a modal-to-amodal trajectory or an early-amodal-representations view. Further, developmental research can be very informative for more general representational theories, particularly concerning the functions that modal and amodal representations play. It thus appears a promising avenue for future research to investigate different issues by exploring the functions and interactions of modal and amodal representations during early childhood and later stages of development. In particular, it seems important that the psychological reality of the two different developmental trajectories be assessed in different cognitive domains.

## Dysfunction

A further important question is whether the representational concepts outlined above can be profitably applied to clinical research to understand dysfunctional behavior and cognition better. Despite their often being neglected, resolving representational issues could be a key to describing dysfunctional behavior and improving treatments for abnormal behavior. In this vein, impulsivity is crucial for a better understanding of many kinds of dysfunctional behavior (Blume et al., 2019; Dawe & Loxton, 2004; Diamond, 2013; Fineberg et al., 2014; Schroeder et al., 2020). Although impulsive behavior often has a strong negative impact on an individual’s life, and on society more broadly, it is still unknown what mechanisms underlie or trigger impulsivity. One possibility is that overly impulsive individuals cannot inhibit behavior triggered by modal representations. Another possibility is that inhibitory issues are generally problematic in impulsive individuals, and that this applies to both modal and amodal representations of objects, events, and situations.

Impairments in inhibitory control are considered a central mechanism in the maintenance of pathological eating behavior such as food-related craving, emotional eating, restrained eating and binge eating (Lavagnino et al., 2016; Wolz et al., 2020, 2021). It is possible that pathological eating behavior is only triggered by modal food representations (e.g., picture-like representations, which are often targeted in food advertisements or experienced with food, such as when passing by the tempting display of a bakery), but that it is not triggered, or triggered less, by amodal food-related representations (e.g., representations given rise by verbal descriptions of a nice meal, the menu in a restaurant; cf. Rumiati & Foroni, 2016). A better understanding of the type of representations that are involved in overeating can lead to improved treatments by revealing their exact relationships with the mechanisms that complicate daily food choices. Such understanding is essential because overeating can be observed in most societies and has already led to pandemic health problems such as overweight and obesity (Ng et al., 2014). In a more general sense, understanding modal and amodal aspects of dysfunctions and disorders can help us understand what is necessary for healthy functioning. In the same vein, the exploration of the effectiveness of if–then plans (i.e., on implementation intentions) that specifically target abstraction processes is of great importance (for research on if–then plans, see Gollwitzer, 1999; see also Gawrilow & Gollwitzer, 2008; Gawrilow et al., 2011). If pathological eating behavior indeed reflects a predominance of modal food representations, then interventions that focus on abstraction processes should be especially effective. To our knowledge, this option has not been investigated in research on pathological eating behavior.

To date there has been little research on the format of representations in research on pathological eating behavior. This is surprising as the view that particular formats are more likely to trigger pathological eating behavior seems to suggest itself. However, first support for this proposal comes from two recent studies using the stop signal task (SST; Logan, 1994; Logan & Cowan, 1984) in which representational format was manipulated indirectly by varying the format of the presented stimuli (pictures vs. words).^12^ Satiated individuals were relatively good at inhibiting pictorial stimuli compared to word stimuli, whereas this was not the case for hungry individuals (however, this difference between the two groups was similar for food- and non-food items; van den Hoek Ostende et al., under review). Although future studies are needed to determine the relevant factors that lead to an increase or decrease of inhibitory control in stimuli of different formats in different groups of participants, this is clearly a promising line of research. An additional complication arises from the possibility that experimentally induced homeostatic states (i.e., hunger and satiety) may be insufficiently sensitive to reveal differences between healthy populations and populations with trait overeating. To this end, restrained eaters may provide a better sample for study because they are characterized by investing *cognitive* effort to restrain food intake despite homeostatic signals of hunger, but also by occasionally losing control over food intake, eventually leading to weight gain (Adams et al., 2019). Indeed, when comparing participants with very high and very low restraint scores (Restraint Scale; Herman & Mack, 1975), only individuals with high restraint scores showed differences in processing pictures and words specific for food pictures. More specifically, for food stimuli, high-restraint individuals were particularly good at inhibiting pictures but not words (Van den Hoek Ostende et al., 2023). This is interesting because it opens the possibility that this group of people strategically upregulates control for a type of stimulus (i.e., pictures) that seems most threatening them, perhaps because these stimuli convey sensorimotor features that trigger pathological eating behavior. Future research is necessary to investigate whether these differences also transfer to actual food intake after processing these types of stimuli.

The above considerations concern the role of modal and amodal representations in the elusive boundary between normal and abnormal behavior as incorporated in the Research Domain Criteria (RDoC) matrix (Insel et al., 2010). However, the functional role of these different representational formats may even be a key to understanding severe clinical disorders. In fact, functional cognitive differences with direct relation to amodal representations have typically been associated with schizophrenia (e.g., Silberstein, 2014). Another example is a newly emerging cognitive approach in autism literature, the “Thinking in pictures” theory (e.g., Bòkkon et al., 2013; Kunda & Goel, 2011; Landgraf & Osterheider, 2013). It hypothesizes that some characteristics which individuals with autism show when solving specific tasks are due to the predominance of modal representations, compared to the more amodal—verbally mediated—approach that individuals without autism use.

Before closing this section, we would like to point out that the distinction between modal and amodal representations is also highly relevant to an emerging research field in clinical psychology, namely the use of virtual reality (VR) setups in researching and treating clinical disorders. Notably, clinical research can highly benefit from VR, as VR environments effectively elicit psychological symptoms (e.g., craving in response to virtual chocolate stimuli; Schroeder et al., 2023b), allowing for causal inferences through highly effective manipulations (e.g., body size manipulations of embodied avatars to measure weight anxiety; Schroeder et al., 2023a). Furthermore, the development of VR applications is also considered a promising tool with increasing potential for the treatment of mental disorders (Freeman et al., 2017; Rizzo & Koenig, 2017). On these grounds, we consider VR not only an optimal method for testing the contribution of modal and amodal processes in mental disorders by enabling the manipulation of stimulus format involving highly photorealistic stimuli as well as their spatial attributes. We are also convinced that insights from research on modal and amodal processes will most likely be fruitful for the development of effective treatment applications in VR. For example, particularly for VR cue exposure and VR-based corrections of attentional biases (Riva et al., 2021), the results of this research can inform future VR studies regarding the required degree of modality in stimulus presentation.

In conclusion, although representational issues seem highly relevant to several areas of clinical psychology, to date only minimal research on the role of different representational formats in human dysfunctions has been conducted. However, some recent studies concerned with inhibitory capacities that indirectly manipulated representational formats by varying the stimulus type indicated that representational issues seem to offer a key to better understanding the mechanism behind pathological eating behavior. In general, we believe that understanding modal and amodal aspects of dysfunctions and disorders can help us understand what is necessary for healthy functioning and pave the way for effective interventions and prevention programs, especially involving the newly emerging VR-based methods.

## Conclusion

The distinction between modal and amodal representations is widely discussed in cognitive psychology, particularly in relation to perception and language. However, it is becoming evident that this differentiation also plays a significant role in other fields of psychology, although receiving less attention than in perception and language. In our review, we have identified three research questions that are crucial for advancing our understanding of representational formats in psychology and related fields. First, it is important to determine the conditions under which cognitive processes rely more on modal or amodal representations. Based on our analysis, we have identified potential factors that may influence the dominance of one representation type over the other, such as the proximity to the object being represented, the frequency of encountering an item, or the age of the individual involved. Second, we need to investigate the functions of modal and amodal representations in cognition. Our analysis reveals that modal representations are well-suited for immediate actions or representing stimuli in close proximity, while amodal representations serve as a basis for comparisons and crossmodal matches. Lastly, we should examine how processes based on modal and amodal cognition interact. Our analysis suggests that certain domains, such as language, learning, and event cognition, are likely to exhibit strong interactions between these two cognitive approaches.

- While our review has predominantly focused on representational formats, it has become apparent that solely considering formats without accounting for cognitive processes is often insufficient. A more comprehensive understanding of cognition necessitates a more explicit examination of both representational formats *and* the cognitive processes that operate on them. Taking an integrated view could provide valuable insights into why different representations are integrated into the cognitive system.
- Our review also shows that the distinction between modal and amodal representations is less sharp than might perhaps have been hoped. However, it is worth bearing in mind that the vagueness of concepts might sometime be beneficial for scientific progress, in that it can inspire new ideas and enable us to see relationships that might otherwise not be evident, allowing us to connect different research fields. We thus agree with William James (1890, p. 6) that the mental is undoubtedly vague and therefore *“it is better not to be pedantic but let the science be as vague as its subject”,* or in Marvin Minsky’s (1988) terms: “*It often does more harm than good to force definitions on things we don’t understand. […] Especially when it comes to understand minds, we still know so little that we can’t be sure our ideas about psychology are even aimed in the right directions. In any case, one must not mistake defining things for knowing what they are.” (p. 39).* Accordingly, we suggest the distinction between modal and amodal representations, despite not being crystal clear, can foster a fruitful exchange of theoretical ideas between domains. This endeavor is undoubtedly made more difficult if each area uses other terms for similar theoretical concepts. What we have sought to show is how overarching principles of different types of representations can be identified, and our hope is that they may contribute to the development of more integrative accounts of human cognition.

## Data Availability

In this manuscript, we do not report original experiments but rather review publications based on experimental results. Therefore, there are no data or materials that could be made available.

## Footnotes

1 As stressed above, it is unclear to what degree all of these dimensions are continuous in a strict sense, especially concerning the dimensions ranging from ‘experiential’ to ‘describing’ and also ‘structure preserved’ to ‘different structure’.

2 Physical dimensions (e.g., sound pressure) that produce experiences (e.g., loudness), which allow quantitative judgments (e.g., “louder than”) are regarded to belong to the *prothetic* domain. By contrast, dimensions (e.g., the wavelength of visible light) that produce experiences (e.g., color) that allow qualitative judgments (e.g., “this apple is green but not red”) are considered phenomena belonging to the *metathetic* domain (Stevens, 1957).

3 One issue which we wish to flag but set aside concerns whether we are realists or instrumentalists about mental representations (Dennett, 1991; Fodor, 1985; Matthews, 2007; Sprevak, 2013). Whilst the issue of realism about mental representations and their features, such as their format, is a vital issue in the philosophy of science, we do not wish to commit ourselves to either side here. We thank an anonymous reviewer for directing our attention to this issue.

4 The philosopher Immanuel Kant (1724–1804), however, claimed that the mind is not decomposable and therefore, among other things, psychology can never become a science. Early psychologists like Wilhelm Wundt, however, challenged his view.

5 From the point of view of optimal encoding, Barlow (1972, p. 371) suggested that “the sensory system is organized to achieve as complete a representation of the sensory stimulus as possible with the minimum number of active neurons” which led the way to combining the advantages of feature and template-based approaches into the theory of “sparse coding” (Olshausen & Field 1996).

6 This effect describes the phenomenon that a letter is easier to recognize within words than in isolation.

7 As noted by Hutchinson and Barrett (2019), the idea of top-down representations is not new. However, there is increasing research that tests this core idea embodied in this predictive coding framework.

8 This interference emerges when the variation of a task-irrelevant dimension (e.g., length of a line) affects the discrimination or classification of stimuli along a task-relevant dimension (e.g., the thickness of the line). In such cases, the two dimensions are not separable but integral dimensions (Garner, 1976).

9 According to this law, the just noticeable difference between two stimulus magnitudes (e.g., two successively lifted weights) is proportional to the smaller stimulus magnitude of the two magnitudes (for a formal definition, see Luce & Galanter, 1963).

10 In this view, when conflicting responses are detected, cognitive control mechanisms are engaged that serve to increase the influence of task-relevant information (e.g., the ink color of a word in the Stroop task) and inhibit the influence of task-irrelevant information (e.g., word’s meaning), helping to resolve the conflict and achieve an agent’s goals. These control mechanisms have recently been implemented in the diffusion model of conflict tasks (Mackenzie & Dudschig, 2021; Ulrich et al., 2015) to account for various conflict adaptation effects (Koob et al., 2022, Koob et al., 2023). More recent versions of the conflict monitoring theory even assume that the monitoring process does not register conflict but detects the affective response (i.e., negative valence, increased arousal) caused by conflict (Dignath et al., 2020, for a review). Thus, on this latter account the relevant representations are assumed to be more modal.

11 Evaluative conditioning occurs when the valence of a stimulus changes as a result of its repeated pairing with another stimulus that carries a positive or negative valence.

12 Although stimulus format is certainly not be confused with the format of the mental representation that they give rise to, we still report these studies here, because they are the only available research in this domain that is related to our debate. We do this based on the assumption that stimuli of different external formats might more readily give rise to one or the other form of mental representation and therefore stimulus format can be considered as an indirect way of manipulating representational format. Thus, although a verbal description and a picture can both in principle lead to a modal or an amodal representation, it still seems to be the case that a verbal description is closer to an amodal representation, and a picture is closer to a modal representation. Thus, it seems plausible to assume that a verbal description can be more readily represented in an amodal representation and a picture more readily in a modal representation.
